# Rifampicin improves neuronal apoptosis in LPS-stimulated co-cultured BV2 cells through inhibition of the TLR-4 pathway

**DOI:** 10.3892/mmr.2014.2480

**Published:** 2014-08-11

**Authors:** WEI BI, LIHONG ZHU, XIUNA JING, ZHIFEN ZENG, YANRAN LIANG, ANDING XU, JUN LIU, SONGHUA XIAO, LIANHONG YANG, QIAOYUN SHI, LI GUO, ENXIANG TAO

**Affiliations:** 1 Department of Neurology, The First Affiliated Hospital of Jinan University, Guangzhou, Guangdong 510632, P.R. China; 2 Department of Pathophysiology, Institute of Brain Research, School of Medicine, Jinan University, Guangzhou, Guangdong 510632, P.R. China; 3 Department of Neurology, Sun Yat-sen Memorial Hospital of Sun Yat-sen University, Guangzhou, Guangdong 510120, P.R. China; 4 Center for Inherited Cardiovascular Disease, Division of Cardiovascular Medicine, Stanford University, School of Medicine, Stanford, CA 94304, USA

**Keywords:** rifampicin, microglia, neuroprotection, TLR-4, neuroinflammation

## Abstract

Agents inhibiting microglial activation are attracting attention as candidate drugs for neuroprotection in neurodegenerative diseases. Recently, researchers have focused on the immunosuppression induced by rifampicin. Our previous study showed that rifampicin inhibits the production of lipopolysaccharide (LPS)-induced pro-inflammatory mediators and improves neuron survival in inflammation; however, the mechanism through which rifampicin inhibits microglial inflammation and its neuroprotective effects are not completely understood. In this study, we examined the effects of rifampicin on morphological changes induced by LPS in murine microglial BV2 cells. Then we investigated, in BV2 microglia, the effects of rifampicin on two signaling pathway componentss stimulated by LPS, the Toll-like receptor-4 (TLR-4) and the nuclear factor-κB (NF-κB). In addition, we co-cultured BV2 microglia and neurons to observe the indirect neuroprotective effects of rifampicin. Rifampicin inhibited LPS-stimulated expression of the *TLR-4* gene. When neurons were co-cultured with LPS-stimulated BV2 microglia, pre-treatment with rifampicin increased neuronal viability and reduced the number of apoptotic cells. Taken together, these findings suggest that rifampicin, with its anti-inflammatory properties, may be a promising agent for the treatment of neurodegenerative diseases.

## Introduction

Microglia, as immune effectors of the central nervous system, respond to pathological conditions and participate in the initiation and progression of neurological disorders such as inflammation and brain tumor, by releasing potential neurotrophic or cytotoxic molecules (1). Increasing evidence indicates that chronic microglial activation may also contribute to the development and progression of neurodegenerative disorders, such as Alzheimer’s disease and Parkinson’s disease (PD), neurotropic viral infections, stroke, paraneoplastic disorders, traumatic brain injury, amyotrophic lateral sclerosis and multiple sclerosis (2–5). Thus, inhibition of activated microglia is an important therapeutic route for neurodegenerative disorders.

Rifampicin is a macrocyclic antibiotic that is extensively used against *Mycobacterium tuberculosis* and other mycobacterial infections (6). The immunosuppressive properties of rifampicin were first reported more than 30 years ago (7–9). Our group previously demonstrated that rifampicin significantly inhibits the lipopolysaccharide (LPS)-induced expression of pro-inflammatory mediators, including inducible nitric oxide (NO), NO synthase (iNOS), cyclooxygenase-2 (COX-2), tumor necrosis factor-α (TNF-α), and interleukin-1β (IL-1β), as well as the production of NO and prostaglandin E_2_ (PGE_2_). Additionally, rifampicin inhibits nuclear factor-κB (NF-κB) via the inhibitor of κB (IκB) pathway. Rifampicin also decreases the phosphorylation of mitogen-activated protein kinases (MAPKs) (10). However, the mechanism through which rifampicin inhibits the production of LPS-induced pro-inflammatory mediators and its neuroprotective effects are not completely understood.

In this study, we investigated the effects of rifampicin on morphological changes induced by LPS in BV2 microglia. Then, we investigated, in murine microglial BV2 cells, the effects of rifampicin on two signaling pathway components stimulated by LPS, the Toll-like receptor-4 (TLR-4) and NF-κB. Our experiments, using the microglia-neuronal co-culture system, demonstrated that rifampicin protects the neurons from microglia-mediated LPS neurotoxicity, supporting that this antibiotic may be effectively used in the prevention of neurodegenerative diseases.

## Materials and methods

### Materials

Rifampicin (purity >98%), LPS, and dimethylsulfoxide were purchased from Sigma-Aldrich (St. Louis, MO, USA). The primary rabbit anti-human polyclonal antibody targeting the NF-κB p65 subunit and the secondary goat anti-rabbit polyclonal rhodamine-conjugated IgG antibody were obtained from Cell Signaling Technology (Beverly, MA, USA). Dulbecco’s modified Eagle’s medium (DMEM) containing L-arginine (200 mg/l), fetal bovine serum (FBS), and other tissue culture reagents were from Gibco^®^ (Thermo Fisher Scientific, Waltham, MA, USA).

### Cell cultures

BV2 immortalized murine microglia were provided by the Cell Culture Center of the Chinese Academy of Medical Sciences (Beijing, China). Cells were cultured in DMEM supplemented with 10% FBS, 100 U/ml penicillin and 100 μg/ml streptomycin in a humidified atmosphere of 5% CO_2_, at 37°C. In all experiments, the BV2 microglial cells were pre-treated with 150 μM rifampicin for 2 h before the addition of LPS (1.0 mg/ml) in serum-free DMEM.

Primary cortical neurons were derived from the cerebral cortices of one-day-old Sprague-Dawley rats that were supplied by the Animal Experimental Center of the Southern Medical University of China [License no. SCXK (yue) 2006–0015]. Animal procedures were performed in accordance with the Guidelines for the Care and Use of Laboratory Animals, which were determined by the Ministry of Science and Technology of China. Primary cortical neurons were derived from the cerebral cortices of 1-day-old Sprague-Dawley rats using previously described procedures (11) with certain modifications: briefly, the isolated tissues were incubated in 0.25% trypsin (Sigma-Aldrich) in phosphate-buffered saline (PBS) for 15 min at 37°C. Following trypsinization, the tissues were rinsed in the Gibco^®^ Neurobasal^TM^ medium containing 2% B27 supplement (both from Thermo Fisher Scientific) three times (5 min each), and were mechanically dissociated using a fire-polished pipette. Cells were seeded at a density of ~2×10^5^ cells/ml on poly-L-lysine (0.1 mg/ml)-coated 24-well culture polystyrene plates and incubated at 37°C with 5% CO_2_, at saturated humidity. After 120 min, cells were gently rinsed three times to remove detached tissues from the surface. Half of the medium was replaced with fresh medium twice a week. Cultures were monitored to ensure that neurons constituted ≥95% of the total population. All experiments were performed using rat primary cortical neurons cultured for 5 days. The primary cortical neurons were verified using an immunofluorescence technique as described in the following section.

### Immunofluorescence staining

For immunofluorescence staining, the cells were fixed with 4% paraformaldehyde for 15 min, permeabilized with 0.1% Triton X-100 for 10 min, and blocked with 5% bovine serum albumin (BSA) for 30 min. The cells were then incubated with the primary antibody targeting NF-κB p65 (1:100 dilution) overnight at 4°C. After washing three times with PBS, the cells were incubated with the secondary antibody for 1 h. Nuclei were stained with Hoechst 33258 (Sigma-Aldrich). Fluorescent images were captured on a laser scanning confocal microscope (LSM 510 META; Carl Zeiss, Stuttgart, Germany).

### Total RNA isolation and reverse transcription-quantitative polymerase chain reaction (RT-qPCR) analysis

Total RNA was isolated with the Invitrogen™ TRIzol reagent (Thermo Fisher Scientific) according to the manufacturer’s instructions. Total RNA (1.0 μg) was reverse transcribed using the M-MLV reverse transcriptase (Promega Corp., Madison, WI, USA) to synthesize complementary DNA. The primers used for qPCR were as follows: TLR-4 forward (F), 5′-GCTTTCACCTCT GCCTTCAC-3′, and reverse (R), 5′-CCAACGGCTCTG AATAAAGTG-3′; 3-phosphate dehydrogenase (GAPDH) F, 5′-TCACCACCATGGAGAAGGC-3′, and R, 5′-GCTAAG CAGTTGGTGGTGCA-3′. qPCR was performed with the following cycling parameters: 40 cycles of denaturation at 94°C for 20 sec, annealing at 62°C for 30 sec, and extension at 72°C for 30 sec. The SYBR-Green qPCR Master Mix 2 kit (Takara Bio Inc., Otsu, Japan) was used in all samples, and the reactions were carried out in a 20 μl final reaction volume, using an LC480 qPCR machine (Roche, Basel, Switzerland). The mRNA expression levels of target genes were calculated based on standard curve analysis with the absolute quantification method, and were expressed relative to the level of *GAPDH*, a housekeeping gene used as an endogenous control (12).

### Cytotoxicity assay in a co-culture of microglia and neurons

The BV2 microglial cells were co-cultured with primary cortical neurons to study the regulation of neuronal survival by the LPS-stimulated microglia. The BV2 microglial cells were grown in Transwell inserts (pore size, 0.4 μm; Corning Life Sciences, Tewksbury, MA, USA), and LPS (1.0 μg/ml) was added. The neurons were then transferred onto the inserts containing BV2 cells. In the Transwell co-culture system, microglial cells communicate with neurons through the semi-permeable membrane without direct cell contact (13). Cell viability was assessed by measurement of released lactate dehydrogenase (LDH), using the CytoTox-96 kit from Promega Corp., according to the manufacturer’s instructions.

### Detection of apoptosis in a co-culture of microglia and neurons

In the co-culture system described above, apoptotic neuronal cells were detected by the terminal deoxynucleotidyl transferase-mediated dUTP nick-end labeling (TUNEL) assay (Roche, Basel, Switzerland). After each treatment, the TUNEL assay was performed according to manufacturer’s instructions, and all nuclei were counterstained with 5 mg/ml of Hoechst 33342 for 10 min at 37°C. The labeled neuronal cells were examined under an LSM 510 META laser scanning confocal microscope. Neuronal cells were considered to be apoptotic when their nuclei were co-stained with Hoechst 33342 and TUNEL. We counted the number of apoptotic cells in 100 randomly chosen neurons, observed at different magnifications.

### Statistical analysis

Quantitative data were expressed as the mean ± standard error of the mean of at least three independent experiments. Comparisons between two groups were analyzed using Student’s t-tests. A value of p<0.05 was considered to indicate statistically significant differences.

## Results

### Effect of rifampicin on morphological changes induced by LPS in BV2 microglia

**Figure 1:**
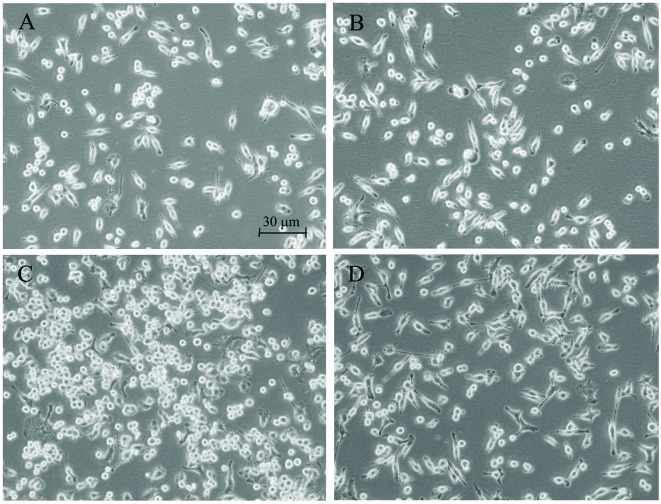
Representative images of microglia incubated for 24 h with fresh medium (control), rifampicin (150 μM) or lipopolysaccharide (LPS; 1 μg/ml). The experiments were performed on three separate microglial preparations. (A) The BV2 microglia shows the typical branching shape at the resting state. (B) Administration of rifampicin causes no obvious morphological changes compared to the non-treated group. (C) Enlargement of the microglial cell body and loss of ramifications, development of an amoeboid shape caused by LPS. (D) Treatment of cells with 150 μM rifampicin markedly improves the morphological changes caused by LPS; the microglia show a branch-like morphology.

Morphological alterations of the microglia are suitable indicators of the effects of different agents. Enlargement of the microglial cell body and loss of ramifications, along with development of an amoeboid shape, are commonly caused by LPS (14). While those changes were clearly observed in the LPS-treated BV2 microglia, rifampicin markedly improved morphological changes that were caused by LPS, and branch-like morphology was observed in these rifampicin-treated cells (Fig. 1).

### Rifampicin inhibits TLR-4 expression in LPS-stimulated BV2 microglia

**Figure 2:**
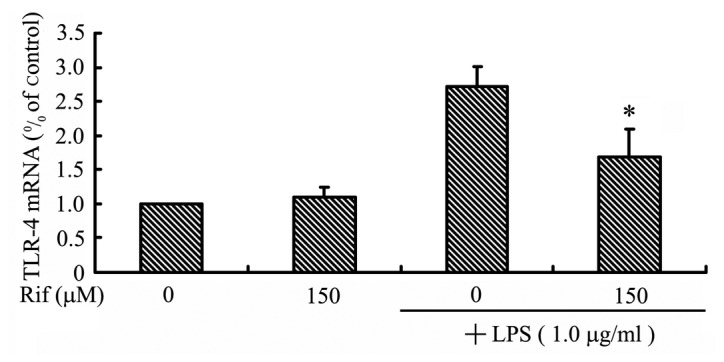
The effects of rifampicin (Rif) on the expression of Toll-like receptor-4 (TLR-4) in lipopolysaccharide (LPS)-stimulated BV2 microglia. Reverse transcription-quantitative polymerase chain reaction analysis of *TLR-4* mRNA expression. Cells were treated with 150 μM rifampicin for 2 h prior to the addition of LPS (1.0 μg/ml) for 1 h. *TLR-4* mRNA levels were calculated relative to the level of the 3-phosphate dehydrogenase (*GADPH*) gene using standard curve analysis. Data were collected from three independent experiments, each carried out in triplicate. ^*^P<0.05 relative to cells treated with LPS in the absence of rifampicin.

To examine the effect of rifampicin on TLR-4 expression, we measured the levels of the *TLR-4* mRNA in LPS-stimulated BV2 microglia. The BV2 microglia were pre-treated with rifampicin for 2 h, and then stimulated with LPS for 2 h prior to RT-qPCR analysis. As shown in Fig. 2, the *TLR-4* mRNA level increased in LPS-stimulated BV2 microglia, but was significantly reduced by treatment with rifampicin. This result indicates that rifampicin inhibits *TLR-4* expression, which we hypothesized may lead to inhibited activation of the NF-κB, MAPK and Akt pathways.

### Effects of rifampicin on the NF-κB signaling pathway

**Figure 3:**
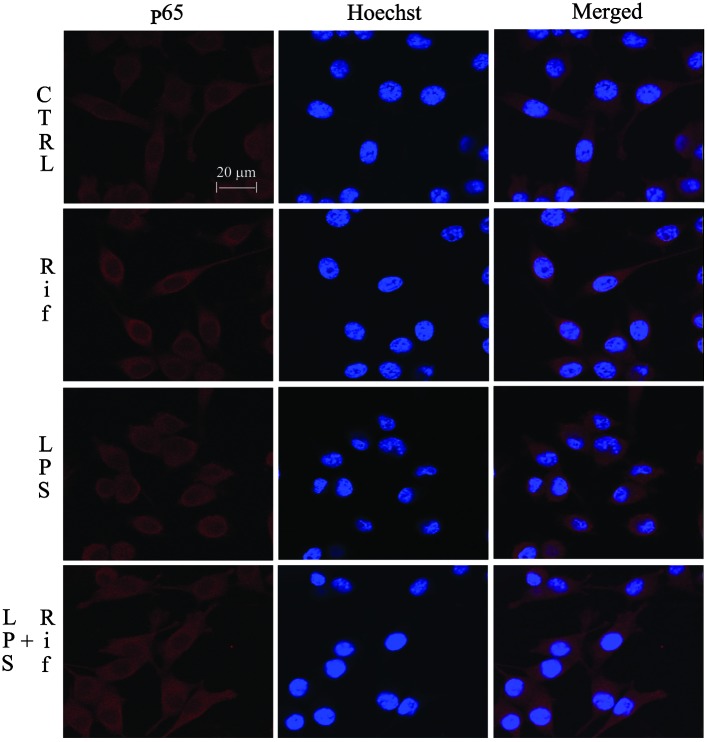
Inhibition of nuclear factor-κB (NF-κB) activation by rifampicin (Rif) in lipopolysaccharide (LPS)-stimulated BV2 microglia. Immunofluorescent staining showing the cellular distribution of the NF-κB p65 subunit (red). Cells were pre-treated with rifampicin (150 μM) for 2 h, followed by LPS treatment (1.0 μg/ml) for 2 h. Hoechst 33258 (blue) was used to visualize the nuclei. Data were collected from three independent experiments, each carried out in triplicate. Merged, double-stained slides; CTRL, control cells.

Activation of NF-κB leads to its translocation to the nucleus, where it mediates the transcriptional regulation of pro-inflammatory genes. The activation and nuclear translocation of NF-κB is a key step in LPS-stimulated microglial activation. We investigated the regulation of NF-κB by rifampicin using immunofluorescence staining. As shown in Fig. 3, the NF-κB p65 subunit was primarily retained in the cytoplasm in unstimulated cells; however, following stimulation with LPS, the cytoplasmic NF-κB p65 level was reduced, accompanied by an increase in the nuclear NF-κB p65 level. Treatment with 150 μM rifampicin blocked NF-κB p65 nuclear translocation in LPS-stimulated BV-2 cells. This result suggests that rifampicin may suppress pro-inflammatory enzymes and pro-inflammatory cytokines by inhibiting NF-κB activation.

### Rifampicin decreases microglial-induced cortical neuron death in a co-culture system

**Figure 4:**
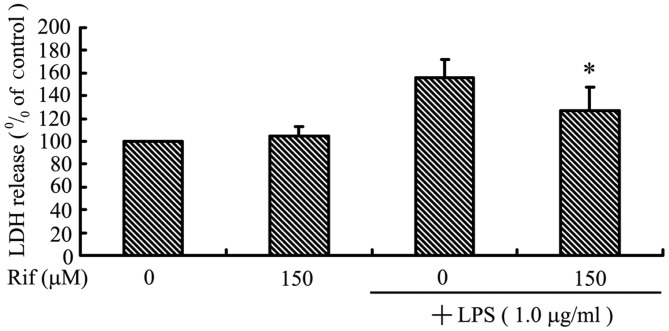

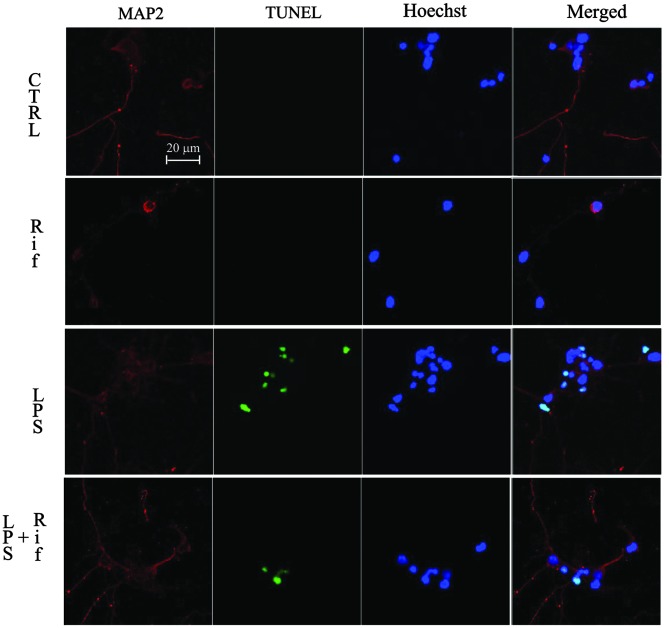

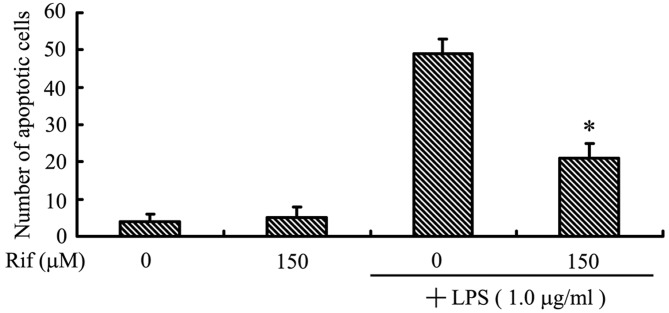
The effect of rifampicin (Rif) on cortical neuron survival in a lipopolysaccharide (LPS)-induced microglial-neuronal co-culture system. Cortical neurons were co-cultured with LPS-activated BV2 microglia with or without pre-treatment with 150 μM rifampicin for 24 h. (A) A lactate dehydrogenase (LDH) assay was used to determine the cortical neuron viability. (B) Immunofluorescent detection of apoptotic cortical neurons co-cultured with BV2 microglia. CTRL, control cells; merged, double-stained slides. (C) The number of apoptotic neurons, counted on double-stained slides from a total of 100 nuclei. Data were collected from three independent experiments, each carried out in triplicate. ^*^p<0.05 compared to cells treated with LPS in the absence of rifampicin.

In order to investigate whether rifampicin can protect against neuronal death induced by microglial activation, we used a co-culture system with cortical neurons and BV2 microglia. We examined cortical neuron viability following co-culture with LPS-activated BV2 microglia using the LDH assay. As shown in Fig. 4A, cortical neurons in control inserts without LPS-stimulated BV2 microglia did not undergo cell death. By contrast, LPS treatment alone led to a high level of cortical neuron death in co-culture, suggesting that LPS-activated microglia secrete pro-inflammatory cytokines that can migrate through the insert, inducing death of the neuronal cells. Treatment with rifampicin markedly reduced the death of cortical neurons: cell viability was increased by ~28.6% when the LPS-stimulated BV2 microglia were pre-treated with rifampicin.

Apoptosis was determined by the TUNEL assay. As shown in Fig. 4B, cortical neurons were stained with Hoechst 33342 (blue), and apoptotic neurons were stained green using the TUNEL method (Fig. 4B). Co-culture with BV2 microglia exposed to LPS alone resulted in a significant increase in the number of apoptotic cortical neurons compared to control cells. As expected, administration of rifampicin reduced the number of apoptotic cortical neurons (Fig. 4C).

## Discussion

Rifampicin has been reported to exert neuroprotective effects in various disease models (15–19). Rifampicin-induced cytoprotection and suppression of β-amyloid aggregation indicate its potential application in the treatment of PD (20,21). *An in vivo* study showed that rifampicin attenuates MPTP-induced neurodegeneration in nigrostriatal dopamine neurons of mouse brains (22). We previously showed that rifampicin pre-treatment causes a dose-dependant increase in cell viability and a reduction in α-synuclein expression (23). Rifampicin-induced neuroprotection was previously attributed to its free radical-scavenging ability (18). We found that rifampicin pre-treatment protects PC12 cells against rotenone-induced cell death. Qualitative and quantitative analysis revealed that rifampicin significantly suppresses rotenone-induced apoptosis by ameliorating mitochondrial oxidative stress (24). We also demonstrated that rifampicin reduces microglial activation and improves neuronal survival during inflammation (10). However, the mechanism through which rifampicin inhibits microglial inflammation and its neuroprotective effects are not completely understood.

Our previous *in vivo* study showed that rifampicin significantly inhibits the LPS-induced expression of pro-inflammatory mediators, including inducible iNOS, COX-2, TNF-α, and IL-1β, as well as the production of NO and PGE_2_ (10). The morphology of microglia cells (Fig. 1), along with data on the expression and synthesis of iNOS, COX-2, TNF-α and IL-1β indicate that rifampicin may induce pro-inflammatory changes in the microglia.

The principal cell surface receptor for the LPS component of antitoxin is TLR-4, member of a highly conserved family of receptors specific to highly conserved bacterial and viral components; these receptors play key roles in activating a cascade of pro-inflammatory events in response to pathogens (25,26). Therefore, treatments that attenuate TLR-4-associated inflammatory cascades may prove beneficial to microglial activation and prevent neurodegenerative processes. Our results indicate that rifampicin pre-treatment inhibits the LPS-induced *TLR-4* expression (Fig. 2). We therefore hypothesized that the underlying molecular mechanism may involve interference with the LPS-triggered increase in *TLR-4* expression. Our previous study showed that rifampicin decreases the phosphorylation of MAPKs (10). Collectively, these results indicate that rifampicin may inhibit NF-κB, p38, JNK, and MAPK activation through downregulation of *TLR-4* expression.

The activation and nuclear translocation of NF-κB is a key step in LPS-stimulated microglial activation, and mediates the transcriptional regulation of pro-inflammatory genes (27,28). In the present study, treatment with 150 μM rifampicin blocked NF-κB p65 nuclear translocation in LPS-stimulated BV-2 cells (Fig. 3). A recent study used a luciferase reporter assay to investigate the possibility that rifampicin inhibits NF-κB transcriptional activity. Further investigation demonstrated that rifampicin blocks the phosphorylation and subsequent degradation of IκB in LPS-induced BV2 cells (10). It is hypothesized that rifampicin markedly inhibits the nuclear translocation of NF-κB p65 (10). These results, also confirmed by the current study, suggest that rifampicin may suppress pro-inflammatory enzymes and pro-inflammatory cytokines through inhibiting the activation of NF-κB.

Microglial activation has been considered harmful for neurons, and can lead to neuronal apoptosis (29). Microglial involvement in neurodegenerative diseases is well-established, microglial activation and neuroinflammation being common features of these neuropathologies (30). Neurotoxic microglial-neuronal interactions have been implicated in the pathogenesis of various neurodegenerative diseases, and have been recognized as critical for the understanding of the underlying mechanism of neuron diseases (31,32). In order to investigate whether rifampicin can rescue neuronal death induced by microglial activation, we used cortical neurons and BV2 microglia in a co-culture system. Our results clearly indicate that when cortical neurons are co-cultured with LPS-stimulated BV2 microglia, neuronal cell death is increased by 56.0%, and the number of apoptotic neurons is increased by 49.0%. However, treatment with rifampicin in our LPS-induced co-culture system increased cell viability by 28.6% and reduced the apoptotic cell number by 28.0%. (Fig. 4). These data suggest that rifampicin decreases cortical neuron apoptosis through inhibition of microglial activation in the microglial-neuronal co-culture system. Together, these results provide strong evidence that rifampicin can protect neurons from microglial-mediated LPS neurotoxicity.

In conclusion, the present study demonstrated that rifampicin inhibits the LPS-stimulated expression of *TLR-4*. When cortical neurons were co-cultured with LPS-stimulated BV2 microglia, pre-treatment with rifampicin increased neuronal viability and reduced the number of apoptotic cells. Our observations suggest that rifampicin may be used as a therapeutic agent for the treatment of neurodegenerative diseases.
